# Case Report: Siblings with neonatal lupus erythematosus

**DOI:** 10.3389/fped.2025.1518881

**Published:** 2025-03-03

**Authors:** Pengyue Tang, Huan Zhang, Ping Li

**Affiliations:** ^1^ Department of Dermatology, Shenzhen Children's Hospital, Shenzhen, China; ^2^ Department of Pathology, Shenzhen Children’s Hospital, Shenzhen, China

**Keywords:** neonatal lupus erythematosus, cutaneous manifestations, siblings, infant, Sjögren's syndrome

## Abstract

Neonatal lupus erythematosus (NLE) is a rare autoimmune disorder characterized by cutaneous and/or cardiac manifestations resulting from the transplacental passage of maternal antibodies, including anti-SSA/Ro, anti-SSB/La, and occasionally anti-U1RNP. This report describes two siblings with distinct NLE presentations, emphasizing the importance of early diagnosis and management, particularly in light of the rising rates of multiple births. A 15-day-old girl (Case 1) presented with classic annular skin lesions and strongly positive SSA and SSB antibodies. Six years later, her brother (Case 2) developed atypical red papules with similar serologic findings. Their mother, diagnosed with Sjögren's syndrome after the first child's (Case 1) presentation, demonstrated suboptimal treatment adherence, which may have contributed to the occurrence of NLE in her second child (Case 2). Neither sibling exhibited systemic involvement, including cardiac manifestations; however, regular monitoring remains essential. These cases highlight the variable NLE phenotype, even within families. In pregnancies with SSA/SSB antibody positivity, close monitoring of antibody titers, electrocardiograms (ECGs), and echocardiograms is paramount for early NLE detection and optimal management, especially given inconsistent maternal treatment. These cases underscore the need for heightened vigilance and proactive strategies in high-risk pregnancies.

## 1 Introduction

Neonatal lupus erythematosus (NLE), first described by Bridge and Foley in 1954, is an autoimmune syndrome caused by the transplacental transmission of maternal autoantibodies, primarily anti-SSA/Ro and anti-SSB/La, and occasionally anti-U1RNP, which bind to fetal tissues (1–3). The incidence of NLE is approximately 1 in 12,500–20,000 live births, with a 2% occurrence in children of mothers carrying these antibodies and an 18%–20% recurrence risk in subsequent pregnancies (3, 4).

Clinically, NLE presents with various manifestations, including the characteristic periorbital annular skin rash (often referred to as an “eye mask” or “raccoon-like” appearance), cytopenia, hepatitis, and, most severely, congenital heart block (CHB) (3, 5).

In NLE, autoantibodies are prevalent in both children and their mothers. Children commonly test positive for anti-SSA (87.5%), anti-SSB (50%), or both (55%), and occasionally for anti-U1RNP (6, 7), while mothers exhibit similar rates: 84.6% for anti-SSA, 58.9% for anti-SSB, and 35.5% for both (8). Additionally, mothers may exhibit other autoantibodies depending on their underlying autoimmune diseases, such as systemic lupus erythematosus (SLE) or Sjögren's syndrome (3, 6).

Diagnosis relies on clinical presentation and detection of anti-SSA/Ro and/or anti-SSB/La in both the infant and mother; skin biopsy is optional (9). Differential diagnoses include congenital syphilis, tinea corporis, sarcoidosis, granuloma annulare, Langerhans histiocytosis, Sweet syndrome, and urticaria (3).

A significant proportion (25%–60%) of mothers are asymptomatic, which poses diagnostic challenges and increases the risk for their offspring (5, 10). Infants may develop other autoimmune diseases later, highlighting the importance of screening women of reproductive age for autoantibodies, even those with mild symptoms (5, 11, 12).

While the global incidence of NLE is a concern, the true incidence is likely underestimated (4). In China, the combination of a large population exceeding 1.4 billion (13) and a high prevalence of autoimmune diseases, such as Sjögren's syndrome (0.33%–0.77%) (14) and SLE (14.09 per 100,000 person-years in 2017) (15), suggests a considerable potential impact of NLE in the country. Furthermore, the implementation of the two-child policy in 2016 may lead to an increase in the incidence of CHB in NLE infants and a rise in overall NLE cases among siblings due to higher numbers of second births (12, 16). These factors collectively highlight the significant public health implications of NLE in China.

Management includes serial fetal echocardiograms biweekly between 16 and 28 weeks' gestation (3, 5), sun protection for cutaneous manifestations (5), and hydroxychloroquine to potentially reduce the recurrence of CHB (17). The role of intravenous immunoglobulin (IVIG) is under investigation (18). The prognosis of NLE varies. Cutaneous, hematologic, and hepatic abnormalities are typically transient. However, CHB can be potentially permanent, particularly third-degree CHB, which is more severe and frequently requires pacemaker implantation due to its significant mortality (20%–30%) and morbidity (19, 20).

These factors underscore the critical need for further research and enhanced surveillance, particularly in countries like China, to better understand and mitigate the impact of NLE.

## 2 Case reports

### 2.1 Case 1

**Figure 1:**
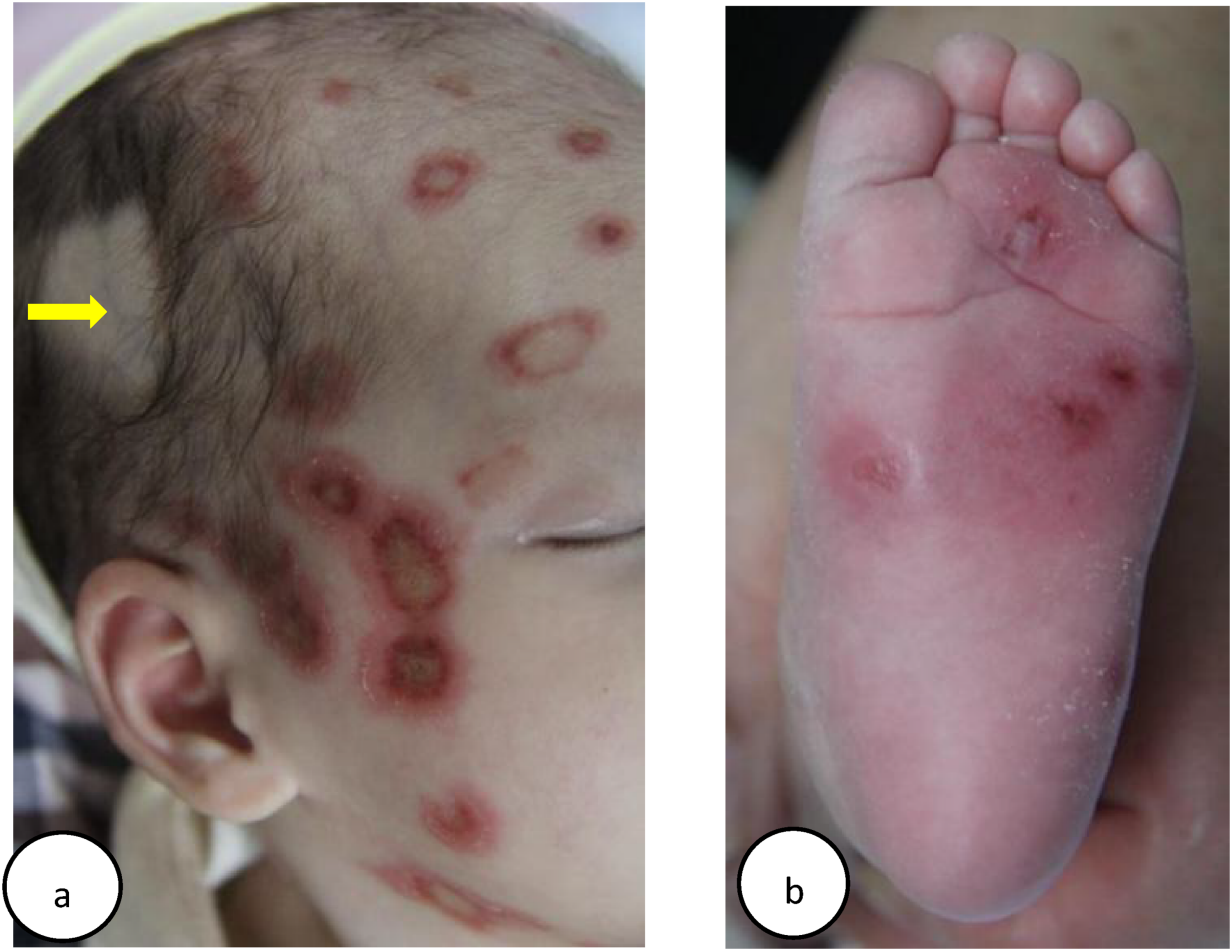
**(a)** Case 1: clinical appearances of head lesions: inflammatory annular plaques, with hyperkeratotic borders and atrophic centers. The alopecia spot on the right side of the head was due to shave by healthcare workers, which could expose the superficial vein of the scalp (yellow arrow), for the purpose of taking blood tests. **(b)** Case 1: multiple discoid skin lesions on the left plantar. Despite their rarity in NLE, plantar lesions are significant indicators of potential systemic involvement, requiring comprehensive evaluations and appropriate management for a favorable prognosis (21).

In October 2015, a 15-day-old girl (patient 1) presented with erythema on her body. Born via vaginal delivery at 40 weeks to a 29-year-old mother, she exhibited targetoid erythematous plaques with central atrophy and raised margins on her face (Figure 1a), trunk, extremities, palms, and soles (Figure 1b). Laboratory tests indicated that the liver and kidney functions and the complete blood count were normal. Serologic tests revealed strongly positive SSA (Ro) and SSB (La) antibodies, a reactive ANA titer of 1:1000 with a speckled nuclear pattern by indirect immunofluorescence (IIF), and a negative syphilis test. Echocardiogram and electrocardiogram (ECG) results were normal. NLE was diagnosed based on the clinical presentation and serologic findings.

Following the infant's positive autoantibody tests, the mother was referred to a rheumatologist. She reported a 2-year history of dry mouth and tested strongly positive for SSA and SSB antibodies, with an ANA titer of >1:1000 and a speckled nuclear pattern by IIF. Schirmer's test indicated reduced tear production in both eyes (the right eye at <5 mm/5 min and the left eye at 5 mm/5 min), and her labial salivary gland biopsy showed focal lymphocytic sialadenitis, characterized by preserved lobular architecture, ductal dilation, and at least three lymphocytic foci per 4 mm^2^. These findings confirmed a diagnosis of Sjögren's syndrome.

The mother was treated with prednisone (20 mg/day for 3 years, then 10 mg/day maintenance) and hydroxychloroquine (400 mg/day), but she showed poor adherence and irregular monitoring of antibody levels.

The infant's rash resolved spontaneously within 6 months with sun protection, and serologic tests for ANA, SSA, and SSB were negative at the age of 1 year. However, residual telangiectasias and hyperpigmentation were observed 8 years later. Notably, the mother's Sjögren's syndrome diagnosis was made after the infant's NLE presentation.

### 2.2 Case 2

Six years later, the mother became pregnant again. At 4 weeks of gestation, serologic tests revealed strongly positive SSA and SSB antibodies, a reactive ANA (>1:1000) with a speckled nuclear pattern (as determined by IIF), consistent with findings from 6 years prior, and an elevated erythrocyte sedimentation rate (ESR) of 90 mm/h (normal <10 mm/h). The mother was prescribed prednisone (10 mg/day), hydroxychloroquine (400 mg/day), and calcium carbonate (600 mg/day) due to her active condition. However, she poorly adhered to the regimen, stopping medications at 20 and 35 weeks of pregnancy due to a cold. Missed doses limited medication use during pregnancy to <20 weeks. At the 32nd week of gestation, her oral glucose tolerance test (OGTT) was normal, but recommended prenatal screenings, such as fetal echocardiograms, were inconsistently performed due to non-adherence.

A male infant (patient 2) was delivered via cesarean section at 39 weeks' gestation due to fetal macrosomia, with a birth weight of 4,500 g, and normal fasting blood glucose (5.1 mmol/L) in the newborn. The ECG result of patient 2 at birth indicated sinus tachycardia, with atrial and ventricular rates of 166 bpm, a P-R interval of 100 ms, and a QRS duration of 77 ms. Although this heart rate was slightly above the normal range for newborns (120–160 bpm) (22), subsequent monitoring revealed stabilization at 140 bpm without the development of CHB.

**Figure 2:**
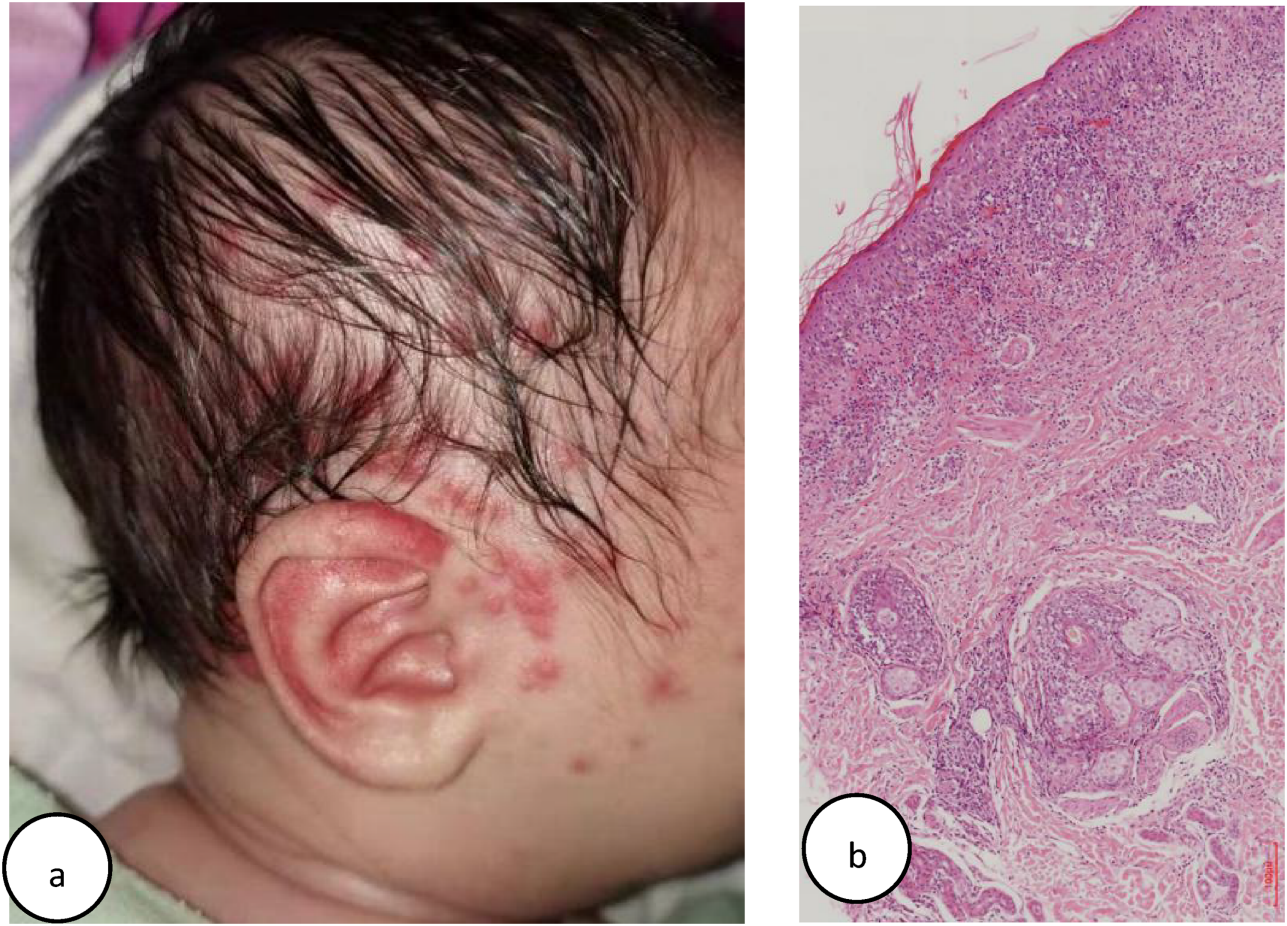
**(a)** Case 2: multiple erythema and papules on one face. **(b)** Case 2: histology of lesional skin: slight epidermal atrophy and hyperkeratosis, vacuolar degeneration at the dermal-epidermal junction, perivascular and periadnexal mononuclear infiltrate in the dermis (Hematoxylin–eosin stain, original magnifification, ×100).

At 1 week after birth, he gradually developed a localized rash on the scalp and face, characterized by erythema and papules (Figure 2a), distinct from his sister's (Case1) widespread targetoid plaques. Laboratory tests revealed positive SSA, SSB, and ANA (1:100) with a speckled nuclear pattern, as determined by IIF. Skin biopsy revealed a vacuolar change of epidermal basal cells and perivascular and periadnexal mononuclear infiltrate in the dermis (Figure 2b). A diagnosis of NLE for Case 2 was made.

In Case 2, the rash resolved by 5.5 months without treatment, with no residual pigmentation or telangiectasia. At 8 months, ANA and SSB were negative, with transient Anti-dsDNA elevation (161 IU/ml), which later turned negative after 2 years. At present, both siblings remain free of skin lesions and autoimmune diseases, under ongoing follow-up.

Consent for publication of this case report and accompanying images was obtained from the patient's parents after they were fully informed and had provided written authorization.

## 3 Discussion

The main results of this study highlight the phenotypic diversity of NLE, as evidenced by the varied dermatological manifestations in siblings, including typical annular plaques and atypical red papules. The study's strengths lie in its comprehensive clinical documentation and extended follow-up, providing valuable insights into the natural history and treatment approaches for NLE in sibling pairs. These findings underscore the critical importance of early diagnosis and treatment.

Comparison with the literature reveals that cases of NLE among siblings have been reported, highlighting the variable clinical presentations of NLE within families. This emphasizes the importance of monitoring and preventive measures for mothers with a history of autoimmune diseases and their offspring. Zuppa et al. (23) discuss the differing manifestations of NLE among siblings, while Lee et al. (24) focus on the increased risk of NLE in successive pregnancies for mothers who have previously given birth to a child with NLE. Collectively, these studies underscore the need for vigilant prenatal care and counseling for families with a history of NLE.

The morphology of the NLE rash was heterogeneous, with typical cutaneous lesions resembling those seen in subacute cutaneous lupus erythematosus (SCLE) (3). A retrospective cohort study (25) revealed that annular (70.7%) and papulosquamous (70.7%) rashes were common, while pink/red macules were less frequent (11.1%).

Our findings align with the literature. Case 1 showed typical annular scaly erythema similar to SCLE, while Case 2 had less common red papules and macules, potentially due to varying specificity of anti-Ro autoantibodies or other fetal-maternal factors, including environmental, intrauterine, or genetic influences (23, 26).

The association between maternal treatment adherence and NLE recurrence requires further empirical evidence. However, it is well-recognized that pregnant women testing positive for autoantibodies to SSA/Ro or SSB/La, particularly those with a prior history of NLE, are at increased risk of delivering a child with NLE (4, 6). Furthermore, recent research has highlighted the notable possibility that normal sinus rhythm can progress to third-degree heart block within just 7 days (19). Given these risks, it is essential to monitor the fetal PR interval through echocardiogram during gestation. The absence of such monitoring is deemed potentially hazardous, underscoring the necessity for comprehensive counseling, fetal and maternal screening, and the implementation of preventive or management strategies for heart disease (6, 27). Our findings align with these observations, suggesting that suboptimal adherence to treatment, which may lead to sustained high levels of autoantibodies such as SSA, SSB, and ANA, could contribute to the recurrence of NLE in subsequent pregnancies. This underscores the crucial importance of strict adherence to treatment protocols to minimize recurrence risk and enhance patient outcomes (19).

Preventing NLE in siblings is challenging due to its genetic and autoimmune etiologies. Key prevention strategies include maternal education on autoimmune disease risks and genetic counseling. In our case, the mother, who had experienced xerostomia for over 2 years, only sought medical attention after her first child was diagnosed with NLE. Despite being diagnosed with Sjögren's syndrome and prescribed treatments, her suboptimal adherence to medication may have contributed to the recurrence of NLE in her second child. This underscores the importance of educating mothers about autoimmune disease risks during pregnancy and the necessity of regular prenatal care and autoantibody monitoring (23, 26). Genetic counseling is recommended for families with a history of NLE to discuss the implications of maternal autoimmune diseases and the associated recurrence risks (26). These measures are crucial for informing clinical practice and research, thereby improving outcomes for families affected by NLE (23).

The study is limited by its small sample size, which may restrict the generalizability of the findings. Additionally, the reliance on parental adherence to treatment introduces variability in disease outcomes, as evidenced by the differing levels of maternal treatment adherence in our two cases. Future studies with larger cohorts and standardized treatment protocols are necessary to address these limitations and provide more robust data on NLE management and outcomes (8).

A retrospective cohort study (25) reported cutaneous sequelae in 34% of 106 patients at a mean follow-up duration of 4 years (range 0.5–18.7 years), including 13% telangiectasia, 17% dyspigmentation, and 9% atrophic scarring. Our findings are consistent with these observations: Case 1 exhibited telangiectasia and hyperpigmentation following rash regression, while Case 2 showed no sequelae. The variability in follow-up durations underscores the importance of long-term monitoring to better understand the disease's natural history and long-term outcomes (27).

## 4 Conclusion

These sibling cases of NLE with distinct cutaneous manifestations highlight the heterogeneous nature of the disease and underscore the importance of early diagnosis and management. Maternal treatment adherence is crucial for preventing recurrence. Long-term follow-up, including cardiac monitoring, is essential even in the absence of initial cardiac manifestations. These findings emphasize the need for increased awareness among healthcare professionals and expectant mothers about NLE, its potential complications, and the importance of preventive strategies. Further research is needed to explore the underlying mechanisms of NLE and to develop more effective prevention and treatment strategies.

## Data Availability

The original contributions presented in the study are included in the article/Supplementary Material, further inquiries can be directed to the corresponding author.
